# Comparison of vasopressin-first weaning versus norepinephrine-first weaning in critically ill patients 

**DOI:** 10.5414/CP204891 

**Published:** 2025-12-05

**Authors:** Maram Alshreef, Hanin AbaAlkhayl, Qoot Almdainy, Abdulaziz Alshammari, Shahad Alajmi, Shatha Alruwaite, Ebtisam Alqahtani, Reema Almalke, Tagreed Alonazi

**Affiliations:** 1 Critical Care Clinical Pharmacist, Pharmaceuticals Service at Prince Sultan Military Medical City (PSMMC), Riyadh,; 2 King Saud Hospital, Pharmaceuticals Service, Qassim,; 3 Critical Care Clinical Pharmacist, Pharmaceuticals Service at Prince Sultan Military Medical City (PSMMC), Riyadh,; 4 University of Hail, Pharmaceuticals Service, Hail,; 5 College of Pharmacy, Almaarefa University,; 6 Pharmacist, King Saud University,; 7 College of Pharmacy, King Saud University, and; 8 College of Pharmacy, Almareefa University, Riyadh, Saudi Arabia

**Keywords:** septic shock, vasopressin, norepinephrine, vasopressor weaning, mechanical ventilation, critical care, mortality

## Abstract

Background: Septic shock is a critical condition requiring vasopressor support and mechanical ventilation. The sequence of vasopressor weaning may affect clinical outcomes, such as mechanical ventilation duration and patient survival. Objectives: This study assesses how vasopressor weaning order affects hemodynamic stability, clinical outcomes, and the length of mechanical ventilation in critically ill septic shock patients. Materials and methods: A retrospective cohort study was conducted at Prince Sultan Military Medical City, Riyadh, Saudi Arabia, from January 2022 to December 2023. Critically ill adult patients receiving intravenous norepinephrine and vasopressin for septic shock and requiring mechanical ventilation were included. Patients were classified into two groups: vasopressin weaned first or norepinephrine weaned first. The duration of mechanical ventilation was the main outcome. These were secondary outcomes: mean arterial pressure (MAP) stability, 30-day and in-hospital mortality, length of stay (LOS) in the intensive care unit and hospital, and rates of reintubation. Results: Among 100 patients (mean age: 65.1 ± 19.7 years; 58% male), vasopressin was weaned first in 47 patients (47%) and norepinephrine first in 53 (53%). Patients extubated while on vasopressors (vasopressin weaned first) had a shorter median duration of mechanical ventilation (4 days) and lower odds of mortality (adjusted OR = 0.30, 95% CI: 0.09 – 0.98; p = 0.046) compared to those weaned off norepinephrine first. No significant differences were observed in reintubation rates or LOS. Conclusion: Weaning vasopressin before norepinephrine may be associated with improved survival and reduced mechanical ventilation duration in septic shock patients, although further research is needed to validate these findings and optimize vasopressor weaning strategies.

**What is known about this subject**

- Currently, evidence shows that discontinuing norepinephrine before vasopressin may result in less frequent episodes of clinically significant hypotension, although this strategy and the reverse strategy (vasopressin-first weaning) show no difference in patient outcome, such as mortality, ICU length of stay, or hospital length of stay. 

**What the study adds**

- This is the first study that examines the effect of weaning strategy for Norepinephrine versus vasopressin with the duration of mechanical intubation. 

## Introduction 

Sepsis is a life-threatening condition characterized by organ failure resulting from dysregulated body response to infection [1, 2]. The progression of sepsis, often due to insufficiency in circulation and hypovolemia, results in septic shock, a type of vasodilatory shock marked by disturbance in systemic vascular resistance and mean arterial pressure (MAP) [3]. Microcirculatory dysregulation in septic shock results in cellular hypoxia and inadequate tissue perfusion, caused by an imbalance between oxygen delivery and demand [4]. About 10% of the intensive care unit (ICU) admissions are for septic shock, and there is a high mortality, around 50% [2, 3, 4, 5, 6, 7, 8]. 

Managing septic shock involves managing the underlying illness, administering fluid resuscitation, and starting vasoactive medication to improve blood pressure and perfusion [9, 10]. Vasoactive medications function by increasing vascular resistance, MAP, and tissue perfusion [1, 2, 3, 4, 5, 6, 7, 8, 9, 10, 11]. For patients suffering from septic shock, norepinephrine should be used as a first-line vasoactive drug, under the Surviving Sepsis Campaign and the International Guidelines for Management of Sepsis and Septic Shock 2021. Its goal is to sustain a target MAP of at least 65 mmHg [6, 7, 8, 9, 10, 11, 12]. When norepinephrine is unavailable, epinephrine or dopamine can be used as an alternative [13]. 

A vital aspect of managing sepsis is oxygen therapy. However, mechanical ventilation is necessary for the majority of patients with acute respiratory distress syndrome or severe lung damage [14, 15]. Although mechanical ventilation is essential for ICU patients, it is associated with complications like increased risk of pneumonia, airway trauma, and stress ulcers, which may result in bleeding and mortality [16, 17, 18]. Liberation from mechanical ventilation involves the process of gradually reducing ventilatory support [16, 18, 19], a critical step to minimize associated complications. 

Most studies in the literature focus on the association between vasopressor discontinuation and the incidence of hypotension as the primary endpoint [20, 21]. However, one study reported a significant reduction in mechanical ventilation duration when non-catecholamine vasopressors (e.g., vasopressin, pituitrin, terlipressin, selepressin, or angiotensin II) were used in conjunction with norepinephrine, compared to norepinephrine alone (p < 0.01) [22]. 

The objective of this study was to investigate whether the sequence in which vasopressors are discontinued influences how long critically ill patients require mechanical ventilation. 

## Materials and methods 

### Study design 

This retrospective cohort study was conducted at Prince Sultan Military Medical City (PSMMC) in Riyadh, Saudi Arabia, a tertiary care hospital with multiple ICUs providing care for medical, surgical, trauma, and burn patients. The ICUs function as closed services with 24/7 onsite coverage by board-certified intensivists and clinical pharmacists. The analysis was conducted on all critically ill patients who received both norepinephrine and vasopressin intravenously (IV) for septic shock during their ICU stay from January 1, 2022, to December 31, 2023. Patients were followed until hospital discharge or died during the in-hospital stay. 

### Ethical consideration 

The study was approved by the Institutional Review Board (IRB) of PSMMC in June 2022. Due to the retrospective and observational nature of the study, informed consent was waived by the PSMMC Research Center Institutional Review Board. All measures were performed following the relevant guidelines and regulations. 

### Study population 

Adult patients aged 18 years or older who received IV norepinephrine and vasopressin for septic shock and required mechanical ventilation during their ICU stay were included in the study. Exclusion criteria comprised patients with COVID-19, receiving vasopressin for cirrhotic complication, ICU stay of less than 24 hours, and death within the first 24 hours of ICU admission. Patients were classified into two groups based on the sequence of vasopressor weaning: 

- Group 1: Vasopressin weaned first, followed by norepinephrine; 
- Group 2: Norepinephrine weaned first, followed by vasopressin. 

These two pathways are used at PSMMC based on clinician preference. 

### Sample size 

A total of 100 patients fit the inclusion criteria and recruited for this study. 

### Data collection 

Data for all eligible patients were collected and retrieved from the hospital electronic medical records system (Rabet). Demographics, comorbidities, vital signs, and severity scores from the Sequential Organ Failure Assessment (SOFA) and the Acute Physiology and Chronic Health Evaluation II (APACHE II) were among the data gathered for this study. Additional data included ICU admission and extubation dates, timing and sequence of vasopressor initiation and weaning, duration of each therapy, and mechanical ventilation status. Patients were followed up until hospital discharge or until they died during the in-hospital stay. 

### Study outcomes 

When comparing the two vasopressin weaning techniques, the main result was the length of time spent on mechanical ventilation. Hospital length of stay (LOS), ICU LOS, 30-day mortality, and in-hospital mortality were secondary outcomes. 

## Results 

### Demographics 

A total of 100 patients were included in the analysis, with the mean age of 65.14 ± 19.69 years, and 58 patients (58.00%) were male. The most common comorbidities were hypertension, observed in 68 patients (68.00%), followed by diabetes mellitus (58.00%), chronic kidney disease (31.00%), and ischemic heart disease (22.00%). 

### Clinical characteristics and vasopressor liberation 

The mean SOFA score was 8.16 ± 3.97, while the APACHE score averaged 18.73 ± 6.77. the median duration of invasive mechanical ventilation was 5 days (interquartile range 3 – 14). reintubation occurred in 29 patients (29%), and the overall mortality rate was 77% (77 patients). 

Among the patients, 47 (47%) were weaned off vasopressin first during mechanical ventilation weaning, whereas 53 (53%) were weaned off norepinephrine. Patients weaned off vasopressors first at the time of extubation had significantly higher SOFA scores (9.11 ± 3.67 vs 7.32 ± 4.08; p = 0.024) and were more likely to die (89.36 vs 66.04%; p = 0.006). No significant difference was observed between the two groups in term of the duration of vasoactive drugs administration, MAP before and after weaning, or other clinical outcomes (p > 0.05). Table 1 provides a detailed summary of the demographic and clinical characteristics stratified by norepinephrine and vasopressin liberation order. 

### Primary outcome: Duration of invasive mechanical ventilation 

Patients aged ≥ 60 years demonstrated a longer duration of invasive mechanical ventilation; however, statistically no difference was observed (β = 12.60, 95% CI: –5.27 – 30.46; p = 0.164). Similarly, patients extubated while receiving continuous norepinephrine (vasopressin liberated first) had a shorter duration of invasive mechanical ventilation compared to those who were weaned off vasopressin first (β = –4.28, 95% CI: –22.19 – 13.64; p = 0.635). On average, patients weaned off norepinephrine first had a 4-day shorter mechanical ventilation duration. This findings, however, were not statistically significant (Table 2). 

### Secondary outcomes 

**Mean arterial pressure before and after weaning**

**Figure 1.**
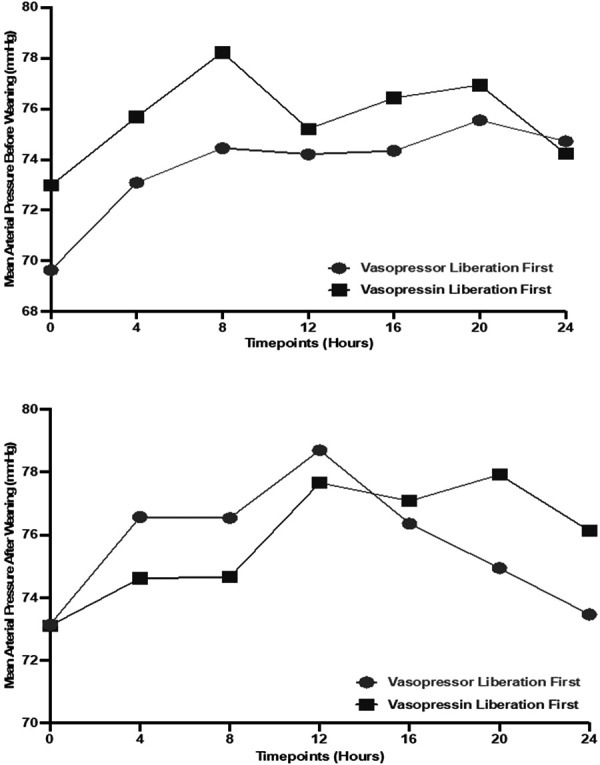
Mean arterial pressure (mmHg) before and after weaning between sequence of norepinephrine vs. vasopressin.

The MAP (mmHg) was assessed at multiple time points (0, 4, 8, 12, 16, 20, and 24 hours) before and after mechanical ventilation weaning. The MAPs were comparable between the two groups (p > 0.05), with both maintaining levels above 65 mmHg. Patients weaned off norepinephrine first exhibited a more stable MAP after weaning compared to those weaned off norepinephrine first, who showed a late downward trend in MAP as shown in Figure 1. 

**Duration of norepinephrine and vasopressin**

**Figure 2.**
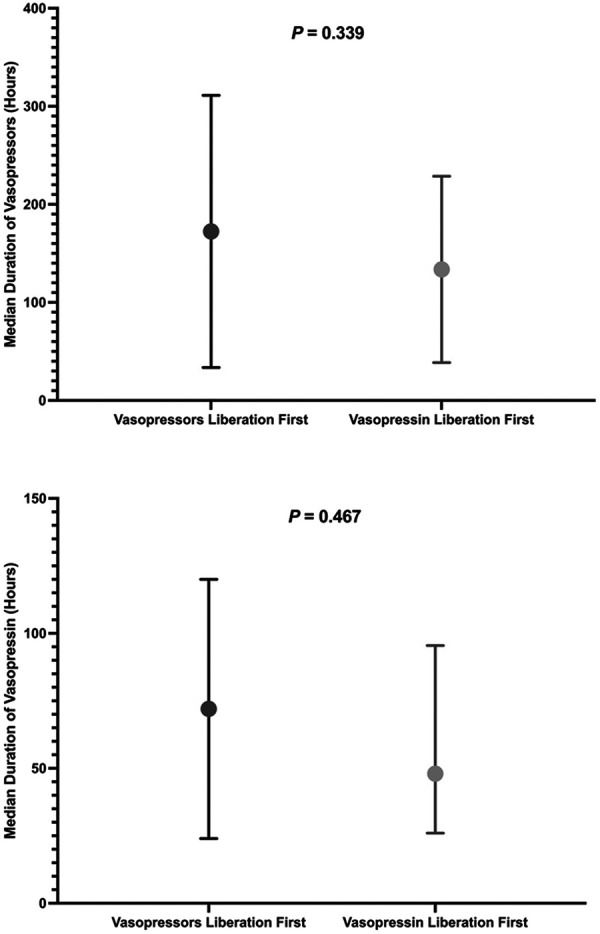
Duration of vasoactive drugs stratified by sequence of norepinephrine and vasopressin liberation order.

The duration of norepinephrine and vasopressin administration was shorter in patients extubated while still receiving norepinephrine (vasopressin liberation first). Median durations were 96 hours (interquartile range: 72 – 168) for norepinephrine and 48 hours (interquartile range: 26 – 95) for vasopressin compared to 120 hours (72 – 264) and 72 hours (24 – 120), respectively, for those weaned off norepinephrine first. These differences were not statistically significant (p = 0.339 and p = 0.467, respectively), as shown in Table 1 and Figure 2. 

**Length of stay in ICU and hospital**

Patients extubated while receiving norepinephrine (vasopressin liberation first) had a shorter length of stay in ICU (β = –4.40, 95% CI: –17.50 – 8.70; p = 0.507) and hospital (β = –6.58, 95% CI: –23.38 – 10.21; p = 0.438) compared to those weaned off norepinephrine first. However, these differences were not statistically significant as shown in Table 3. 

**Reintubation and mortality**

**Figure 3.**
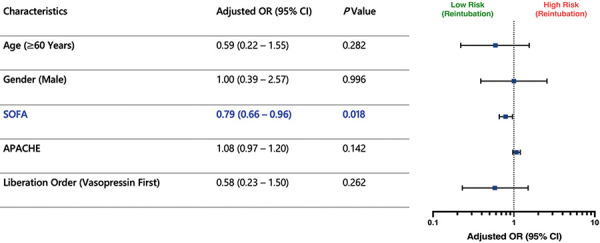
Association of sequence of norepinephrine and vasopressin liberation order with reintubation (multivariable logistic regression). CI = confidence interval; SOFA = Sequential Organ Failure Assessment; APACHE = Acute Physiology and Chronic Health Evaluation.

**Figure 4.**
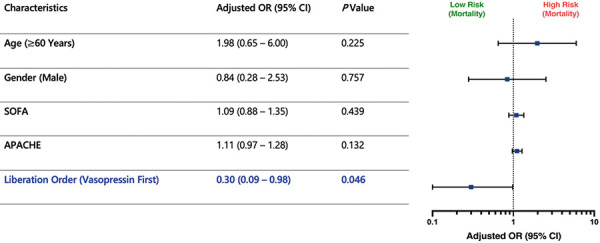
Association of sequence of norepinephrine and vasopressin liberation order with mortality (multivariable logistic regression). CI = confidence interval; SOFA = Sequential Organ Failure Assessment; APACHE = Acute Physiology and Chronic Health Evaluation.

Multivariable logistic regression analysis was used to analyze the relationship between the order of norepinephrine vs. vasopressin discontinuation and outcomes including reintubation and death. Adjusting confounders: including age ≥ 60 years, male gender, SOFA score, and APACHE score, no significant association was observed between norepinephrine liberation order and reintubation (adjusted OR = 0.58, 95% CI: 0.23 – 1.50; p = 0.262) as shown in Figure 3. However, patients extubated while on norepinephrine (vasopressin liberated first) had significantly lower odds of mortality compared to those weaned off vasopressor first (adjusted OR = 0.30, 95% CI: 0.09 – 0.98; i = 0.046), suggesting a possible survival benefit as shown in Figure 4. 

## Discussion 

Vasopressors, such as vasopressin and norepinephrine, are essential for managing hypotension in critically ill patients, particularly those with septic shock [23]. The sequence of vasopressor weaning has been a topic of interest due to its potential impact on hemodynamic stability and clinical outcomes [24]. This study examined the clinical outcomes of weaning vasopressin before norepinephrine compared to the reverse strategy, focusing on the duration of mechanical ventilation, hemodynamic stability, and overall patient outcomes. 

Previous studies have extensively analyzed the incidence of hypotension during vasopressor weaning. A meta-analysis evaluating hypotension within 24 hours of vasopressor discontinuation found a significant reduction in hypotension incidence when norepinephrine was discontinued first (31.8%) compared to vasopressin (58.8%) [25]. 

Cheng et al. [1] (2019) conducted a meta-analysis of 43 randomized controlled studies involving 5,767 septic shock patients to evaluate the effect of different types of vasoactive medications. The primary outcome, 28-day mortality, showed no significant difference across different vasopressor strategies, with consistent findings for ICU mortality (χ^2^ = 13.75, p = 0.68, I2 = 0%). Furthermore, Zhong et al. [22] (2020) assessed the safety and efficacy of combining non-catecholamine vasopressors with norepinephrine. The 28-day death rate was slightly reduced by this combination (p = 0.02), but the 90-day, ICU, and in-hospital mortality rates were unaffected (p > 0.05). A notable finding was a reduction in the length of mechanical ventilation (standard mean difference: −0.19;95% CI: –0.31 to − 0.07; p < 0.01), although no significant effects were observed on ICU or hospital LOS, or continuous renal replacement therapy duration. 

This study provides additional insight into the impact of vasopressor weaning order. Unlike prior studies that primarily focused on hypotension incidence, this research highlighted differences in mortality and duration of mechanical ventilation between the two strategies. Patients weaned off vasopressin first demonstrated a potential survival benefit, with reduced odds of mortality, suggesting that a nuanced approach to vasopressor discontinuation may enhance patient outcomes. 

A significant gap addressed by this study is the limited understanding of how the vasopressor weaning sequence affects the duration of mechanical ventilation and hemodynamic parameters over time. Previous research often failed to account for detailed hemodynamic trends before and after vasopressor discontinuation. By evaluating these parameters longitudinally, this study provides a more comprehensive understanding of weaning strategies’ clinical impact. 

Further validation studies are required, as well as further evaluation of optimal weaning protocols in a variety of different patient populations. Additionally, standardizing vasopressor weaning strategies could reduce variability in practice and improve outcomes in critically ill patients. 

## Conclusion 

This study draws attention to the clinical implications of the order in which vasopressors, specifically vasopressin and norepinephrine, are weaned in critically ill patients. Discontinuing norepinephrine first was associated with a lower incidence of hypotension compared to vasopressin-first weaning, consistent with findings from previous studies. However, this research also underscores that the choice of weaning strategy has significant implications for mortality rates and mechanical ventilation duration. Future research should focus on validating these findings and establishing standardized weaning protocols to optimize care for critically ill patients. 

## Authors’ contributions 

Dr. Maram conceived of the presented idea. Dr. Hanin developed the theory and performed the computations. 

All Authors verified the analytical methods. All authors worked on the data and discussed the results and contributed to the final manuscript. 

## Funding 

This reach received no funding from any agency. 

## Conflict of interest 

The authors declare no conflict of interest relevant to this study. 

**Table 1.** Characteristics of the study patients stratified by sequence of norepinephrine and vasopressin liberation order.

Variables	Total n (%)	Vasopressors liberation first n (%)	Vasopressin liberation first n (%)	p-value
No. of patients	100	47 (47.00%)	53 (53.00%)	–
Age (years, mean ± SD)	65.14 ± 19.69	64.89 ± 20.62	65.36 ± 19.02	0.907
Sex				
Female	42 (42.00%)	16 (34.04%)	26 (49.06%)	0.129
Male	58 (58.00%)	31 (65.96%)	27 (50.94%)	
Comorbidities				
Hypertension	68 (68.00%)	31 (65.96%)	37 (69.81%)	0.680
Diabetes mellitus	58 (58.00%)	26 (55.32%)	32 (60.38%)	0.609
Chronic kidney disease	31 (31.00%)	16 (34.04%)	15 (28.30%)	0.536
Cerebrovascular accident	21 (21.00%)	12 (25.53%)	9 (16.98%)	0.295
Dyslipidemia	16 (16.00%)	10 (21.28%)	6 (11.32%)	0.175
Ischemic heart disease	22 (22.00%)	9 (19.15%)	13 (24.53%)	0.517
Heart failure	16 (16.00%)	10 (21.28%)	6 (11.32%)	0.175
Atrial fibrillation	18 (18.00%)	9 (19.15%)	9 (16.98%)	0.778
Venous thromboembolism	8 (8.00%)	6 (12.77%)	2 (3.77%)	0.143^†^
Renal tubular acidosis	1 (1.00%)	1 (2.13%)	0 (0.00%)	0.470^†^
Disease severity				
SOFA score (mean ± SD)	8.16 ± 3.97	9.11 ± 3.67 20.09 ± 6.44	7.32 ± 4.08 17.53 ± 6.89	0.024 0.059
APACHE score (mean ± SD)	18.73 ± 6.77			
Duration of vasoactive drugs (hours, median (IQR))				
Vasopressors	107(72–192)	120 (72–264)	96 (72–168)	0.339
Vasopressin	48 (26–97.5)	72 (24–120)	48 (26–95)	0.467
MAP (mmHg, mean ± SD)				
Before weaning	74.75 ± 41.42	73.71 ± 32.97	75.67 ± 5.89	0.395
After weaning	75.81 ± 5.16	75.74 ± 5.45	75.88 ± 5.97	0.928
Outcomes				
Duration of IMV (days, median (IQR))	5 (3 – 14) 29 (29.00%)	7 (3 – 15) 15 (31.91%)	5 (4 – 14) 14 (26.42%)	0.850 0.545
Reintubation	18 (7 – 31.5)	18 (6 – 31)	18 (7 – 32)	0.629
Length of stay in ICU (days, median (IQR))	27.5 (14 – 40.5)	28 (13 – 43)	27 (16 – 39)	0.922
Length of stay in hospital (days, median (IQR)) mortality	77 (77.00%)	42 (89.36%)	35 (66.04%)	0.006

SD = standard deviation; SOFA = Sequential Organ Failure Assessment; APACHE = Acute Physiology and Chronic Health Evaluation; IQR = interquartile range; IMV = invasive mechanical ventilation; ICU = intensive care unit; MAP = mean arterial pressure. ^†^Fisher’s exact test.

**Table 2.** Association of sequence of norepinephrine and vasopressin liberation order with duration of invasive mechanical ventilation.

Variables	Coefficient (SE)	95% CI	p-value
Age (≥ 60 years)	12.60 (8.96)	–5.27 – 30.46	0.164
Gender (male)	–9.38 (8.66)	–26.65 – 7.90	0.283
SOFA	0.73 (1.53)	–2.33 – 3.79	0.635
APACHE	–0.51 (0.95)	–2.41 – 1.39	0.591
Liberation order (vasopressin first)	–4.28 (8.98)	–22.19 – 13.64	0.635

SE = standard error; CI = confidence interval; SOFA = Sequential Organ Failure Assessment; APACHE = Acute Physiology and Chronic Health Evaluation.

**Table 3.** Association of sequence of norepinephrine and vasopressin liberation order with length of stay in ICU and hospital.

Variables	Length of stay in ICU			Length of stay in hospital		
	Coefficient (SE)	95% CI	p-value	Coefficient (SE)	95% CI	p-value
Age (≥ 60 years)	2.42 (6.81)	–11.11 – 15.94	0.723	–0.17 (8.73)	–17.51 – 17.17	0.985
Gender (male)	2.48 (6.61)	–10.64 – 15.59	0.709	2.03 (8.47)	–14.80 – 18.83	0.812
SOFA	–0.95 (1.24)	–3.41 – 1.51	0.444	–0.04 (1.59)	–3.20 – 3.11	0.979
APACHE	–0.12 (0.71)	–1.54 – 1.30	0.870	–0.79 (0.92)	–2.61 – 1.03	0.392
Liberation order (vasopressin first)	–4.40 (6.60)	–17.50 – 8.70	0.507	–6.58 (8.46)	–23.38 – 10.21	0.438

ICU = intensive care unit; SE = standard error; CI = confidence interval; SOFA = Sequential Organ Failure Assessment; APACHE = Acute Physiology and Chronic Health Evaluation.
